# Hysteroscopic surgery for treating intramural fibroids at the cesarean scar area: a case report and literature review

**DOI:** 10.3389/fmed.2025.1632322

**Published:** 2025-09-09

**Authors:** Juntong Wu, Yuan Liu, Lili Zhang, Dongji Ma, Tianming Yao, Li Wang

**Affiliations:** ^1^Department of Obstetrics and Gynecology, The 964th Hospital, Changchun, Jilin, China; ^2^Obstetrics and Gynecology Diagnosis and Treatment Center, The Affiliated Hospital, Changchun University of Chinese Medicine, Changchun, Jilin, China; ^3^Department of Cardiovascular Medicine, The 964th Hospital, Changchun, Jilin, China

**Keywords:** experienced surgical team, hysteroscopy, rigorous fluid monitoring, scar site of cesarean section, technical challenges, uterine fibroids

## Abstract

**Background:**

Uterine fibroids located at cesarean section scar sites are rare and differ clinically from conventional fibroids, frequently causing abnormal uterine bleeding and infertility. Traditional surgical approaches (laparotomy, laparoscopy, or vaginal surgery) necessitate incision of the scar site, resulting in significant tissue damage. We present a successful case of hysteroscopic resection for such fibroids, demonstrating the minimally invasive advantages of this approach.

**Case summary:**

A 49-year-old multiparous woman with a history of cesarean delivery presented with a 2-year history of menorrhagia and prolonged menstrual cycles, exacerbated over the preceding month. Gynecological ultrasound revealed heterogeneous hypoechogenicity within the cervical canal, measuring approximately 5.3 cm × 3.5 cm × 3.3 cm in size. The physical examination revealed an enlarged uterus, approximately the size of 11 cm × 8 cm × 7 cm. Hematologic workup showed the hemoglobin (HGB) level of 65 g/L. Based on these examination results, a clinical diagnosis of “uterine fibroids, secondary anemia” was conducted. Hysteroscopy examination showed that the base of the uterine fibroid was attached to the scar site of the original cesarean section, without a pedicle, and the main body of the fibroid remained in the cervical canal—intraoperative definitive diagnosis: intramural fibroids at the scar site of the uterus. Therefore, a hysteroscopic approach with a bipolar resectoscope was used to remove the intramural fibroids in the scar area of the uterus. The procedure consumed 22,000 ml of fluid distension media (0.9% NaCl) and lasted for 1 h and 45 min. The postoperative pathological diagnosis was uterine leiomyoma, consistent with the preoperative diagnosis. A follow-up was conducted 3 months after surgery, and a gynecological ultrasound examination showed complete removal of uterine fibroids.

**Conclusion:**

This case confirmed that hysteroscopic resection of cesarean scar intramural fibroids is a feasible, safe, and minimally invasive approach.

## 1 Introduction

**Figure 1:**
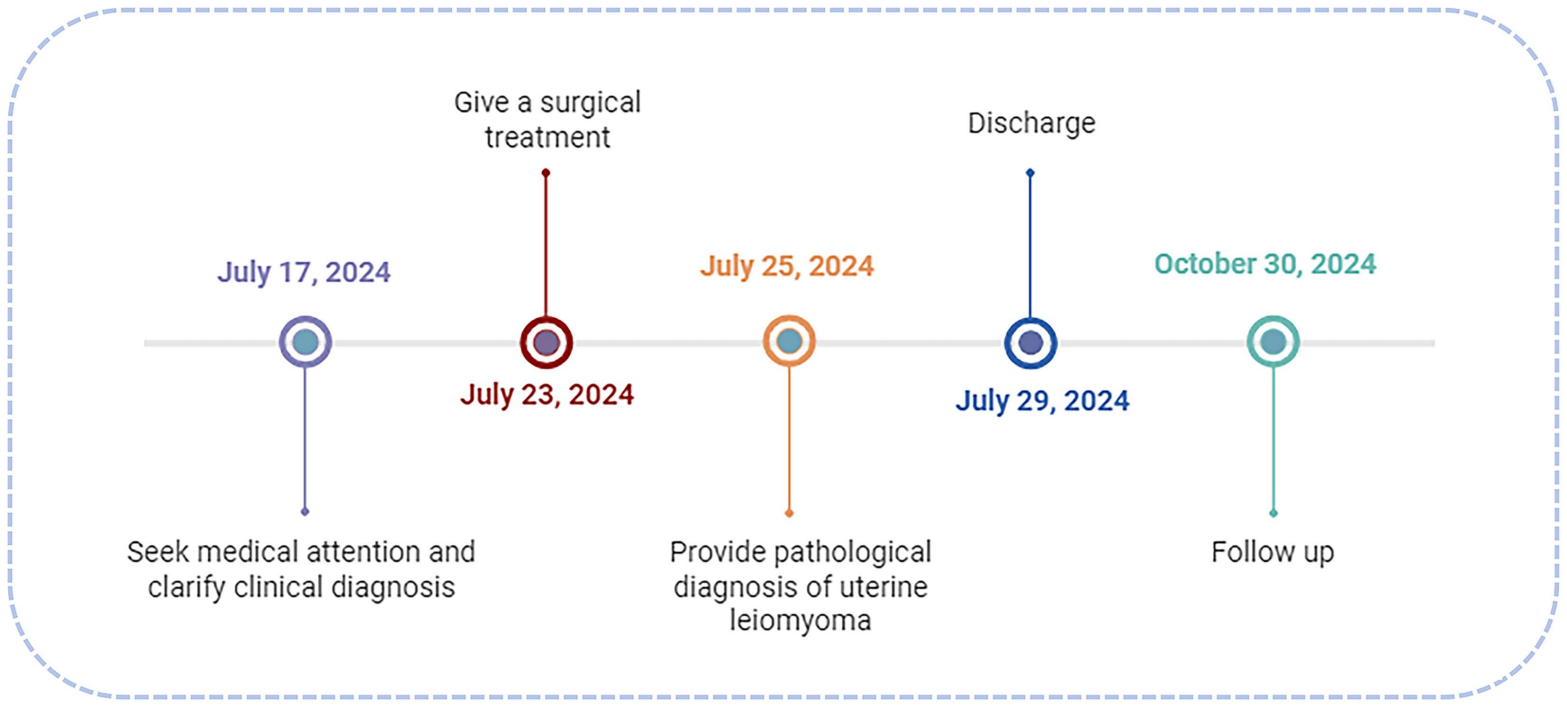
Timeline of historical and current episode of care.

Uterine fibroids arising from cesarean section scars are rare and typically protrude into the uterine cavity. The attenuated myometrium at the scar site impairs contractility, predisposing to menorrhagia and infertility (1). Traditional approaches (laparotomy, laparoscopy, or vaginal surgery) require scar incision, posing risks of hemorrhage and ureteral injury due to proximity to the uterine artery and ureter. Hysteroscopy offers direct visualization for precise resection without abdominal incisions, minimizing ureteral risk (2). In this article, we detailed a hysteroscopic resection of a cesarean scar fibroid and explored its clinical value through a literature review (Figure 1).

## 2 Case presentation

**Figure 2:**
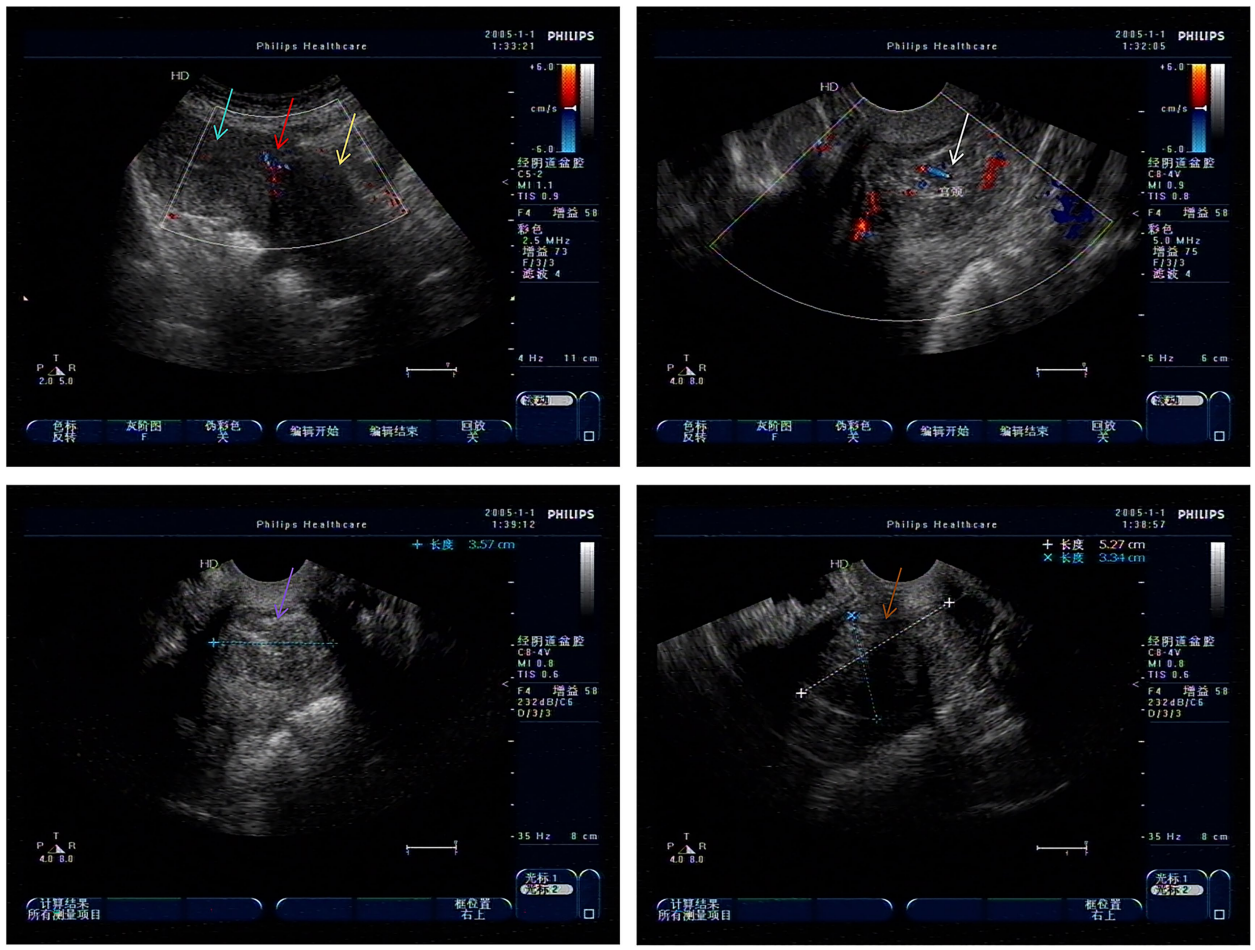
Low echo of cervical canal in gynecological ultrasound examination (Green arrow: uterine body; Red arrow: uterine scar; Yellow arrow: cervical area; White arrow: Cervical hypoechogenicity and blood flow; Purple and brown arrow: Cervical hypoechogenicity).

A 49-year-old G_1_P_1_ (1999 cesarean delivery) presented with 2 years of menorrhagia (10–15 days/cycle) and worsening fatigue. Ultrasound showed a 5.3 cm × 3.5 cm × 3.3 cm heterogeneous cervical mass (Figure 2). The physical examination showed an enlarged uterus, approximately the size of 11 cm × 8 cm × 7 cm. Hematologic workup showed the hemoglobin (HGB) level of 65 g/L. Based on these examination results, a clinical diagnosis of “uterine fibroids, secondary anemia” was conducted. Preoperative preparation was improved by administering 600 mL of red blood cell suspension to correct anemia. After the HGB reached 94 g/L, misoprostol was administered to soften the cervix for 3 days.

**Figure 3:**
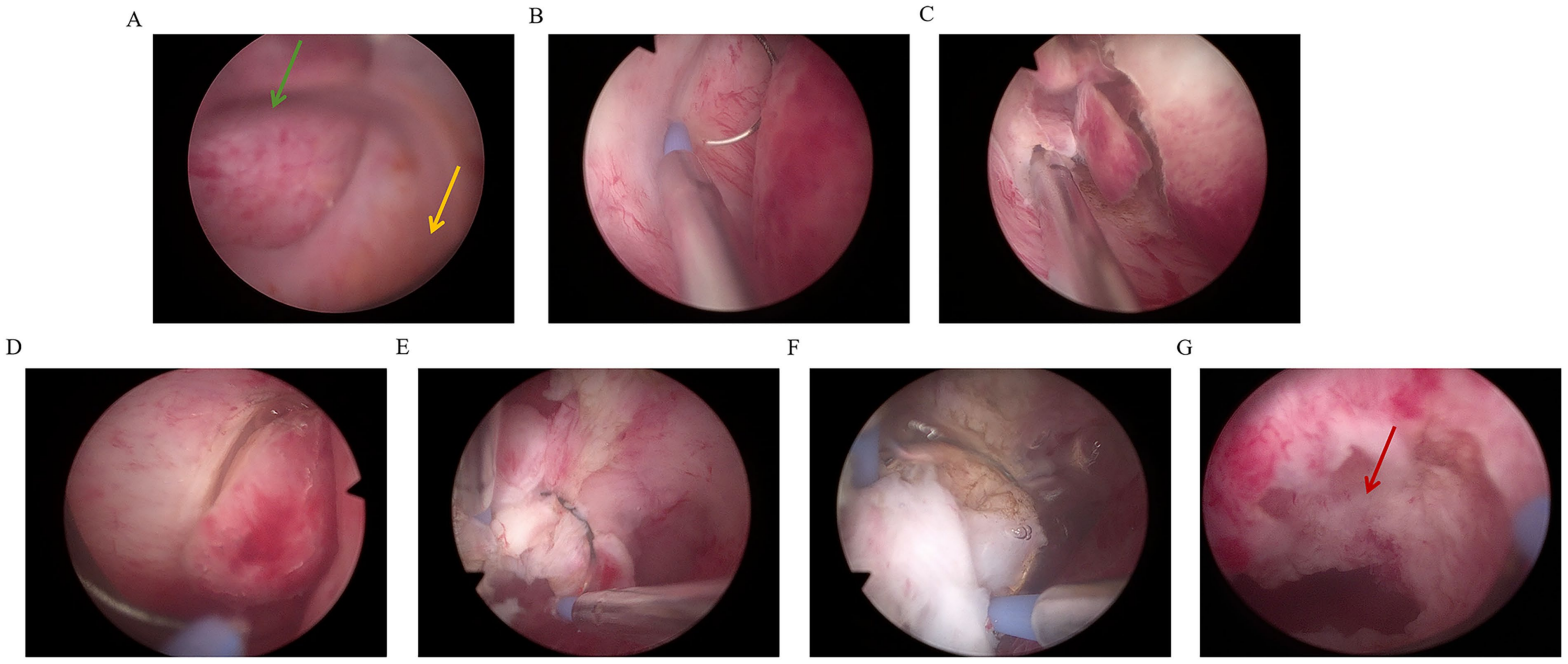
Schematic diagram of excision of intramural fibroids in scar area of cesarean section under hysteroscopy [**(A)** The green arrow represents fibroids, and the yellow arrow represents the external cervical opening; **(B)** Right side fibroid resection; **(C)** left fibroid resection; **(D)** Right side fibroid resection; **(E,F)** Reverse resection of fibroid base; **(G)** The red arrow indicates the location of the cesarean scar at the base of the fibroid].

**Figure 4:**
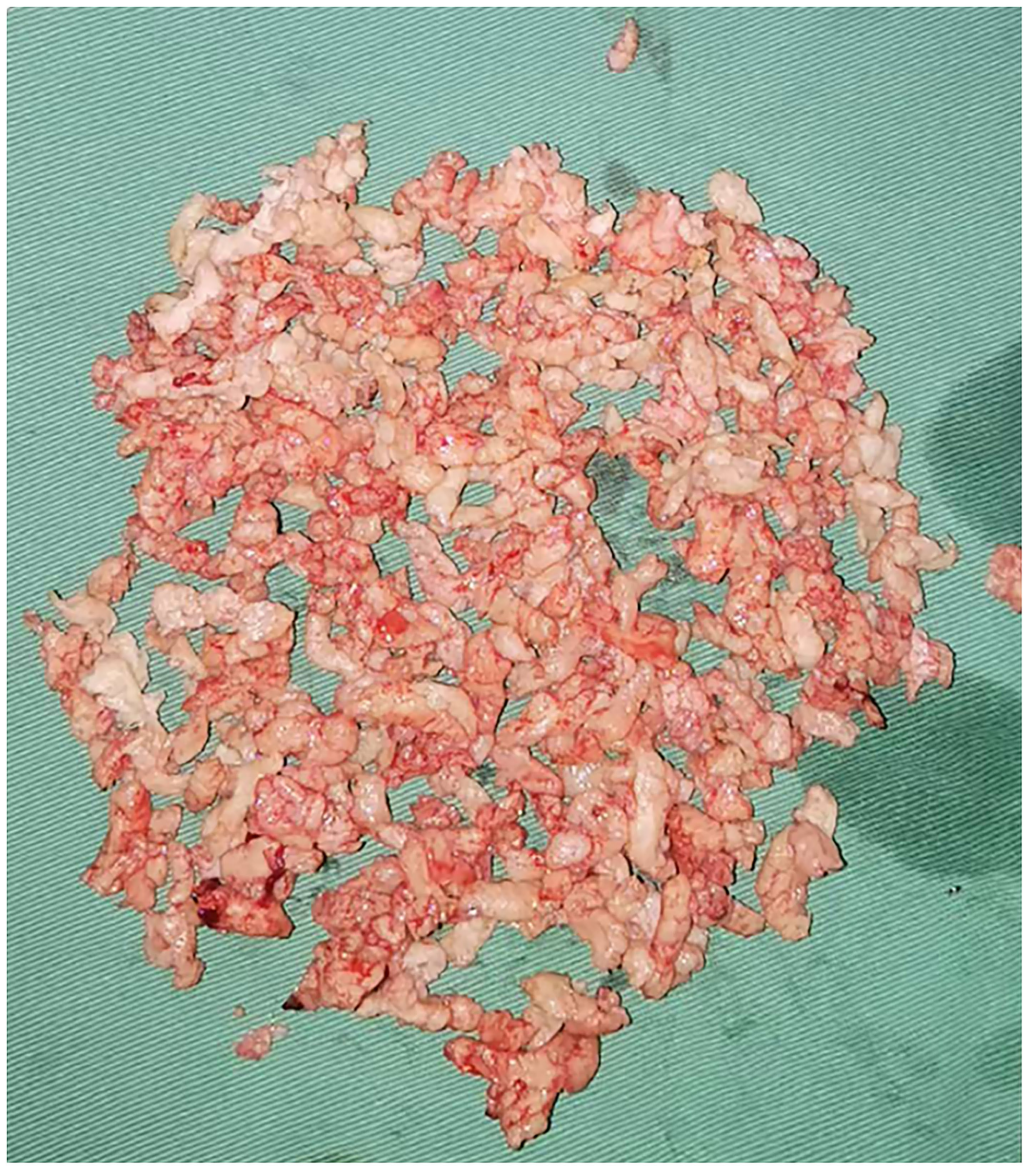
Broken fibroid tissue.

A 0.9% NaCl solution was used as the distension medium, and bipolar electrocautery was performed to remove the fibroids. During the operation, it was observed that the base was attached to the scar of the original cesarean section, and the main body of the fibroid remained within the cervical canal. The specific surgical steps were as follows: Under ultrasound guidance, the fibroid (attached to the scar) was resected using a loop resectoscope in antegrade/retrograde motions. Intermittent intravenous infusion of oxytocin and continuous intravenous infusion of diluted oxytocin (500 mL of balanced solution plus 20 units of oxytocin) were applied. Through continuous electrocautery, the volume of the fibroid gradually decreased, but the fibroid was not squeezed into the uterine cavity. So, the surgery time was extended. One hour after the surgery, the muscle layer at the scar site of the uterus increased from 3 mm to 5 mm. The remaining fibroid was removed along the edge of the fibroid using a reverse cutting method, close to the base, and the fibroid was separated from the scar site using a retrograde electric cutting ring. After the field of view was clearly exposed, especially in the scar area of the uterus, the fibroid was removed completely by the incision method (Figures 3, 4). The surgery required 22,000 mL of distension fluid (plasma liquid recovered from medical plastic buckets, combined with negative pressure suction bottle measurement, estimated to be approximately 21,000 mL; Supplementary Figure S1), with a duration of 1 h and 45 min. Furosemide (10 mg) prevented fluid overload. Blood gas/electrolytes remained normal. Pathology confirmed uterine leiomyoma. Three-month follow-up demonstrated complete resolution.

## 3 Discussion

For fibroids located in the lower uterine segment, treatment options include hysteroscopic surgery, focused ultrasound, transabdominal or laparoscopic myomectomy, and total hysterectomy. Hysteroscopic surgery, a natural orifice procedure, is particularly suitable for fibroids protruding into the uterine cavity. It offers minimal tissue damage, no external incisions, and reduced risk to the ureter and uterine artery, making it ideal for patients seeking fertility preservation or organ conservation. However, limitations include the need for staged resection with large fibroids, the risk of fluid overload, and higher recurrence rates (3, 4). Focused ultrasound therapy provides rapid recovery and fewer complications but often requires multiple sessions and has a high recurrence rate (5). Transabdominal/laparoscopic myomectomy is preferred for cervical fibroids extending to the broad ligament. This approach carries surgical challenges due to the complex anatomy, with potential risks of ureteral or bladder injury (6). Transvaginal surgery involves posterior fornix resection and serosal incision, facing issues such as limited field of view, complex operation, high blood loss, and ureteral injury (7). Total hysterectomy is a curative treatment, but patients suffer from loss of fertility and severe perimenopausal syndrome caused by disrupted ovarian blood supply and imbalanced hormone secretion (8, 9). This 49-year-old patient strongly desired uterine preservation despite no fertility requirements. Considering the characteristics of its fibroids protruding toward the uterine cavity and the demand for quality of life, it was recommended to prioritize hysteroscopic surgery.

Intraoperative hysteroscopic evaluation identified a fibroid exceeding 5 cm in diameter with its base anchored at the cesarean scar site, which cannot be seen from the external cervical opening. This anatomical presentation introduced unique technical challenges, particularly concerning the safe and complete resection of the fibroid. The proximity of the fibroid to the scarred tissue, combined with its low cervical position, necessitated meticulous surgical planning and execution. Such complex cases underscore the importance of surgical expertise in hysteroscopic management, as operative success heavily relies on the surgeon’s proficiency in navigating anatomical constraints and mitigating potential complications.

Previous literature has demonstrated that surgeons with extensive experience in hysteroscopic procedures exhibit a significantly lower risk of complications during technically demanding surgeries compared to their less-experienced counterparts (10). This underscores the critical role of surgical proficiency in achieving successful outcomes. In the present case, the fibroid was located in the scar area of the cesarean section, at a low position, which posed unique challenges. During hysteroscopy, the continuous outflow of distension fluid through the cervix substantially reduced the uterine distension effect, a scenario distinct from cases involving fibroids located within the uterine cavity. Upon endoscopic visualization, the fibroid base was identified at the cesarean section scar site, presenting as a sessile lesion. Notably, the myometrial layer at this region is markedly thinner than normal uterine tissue, with compromised elastic properties and diminished contractility (11, 12). These anatomical alterations represent key risk factors for hemorrhage during cesarean scar-related procedures (13). Conventional hysteroscopic resection techniques, including en bloc resection, fibroid morcellation, grasping, twisting, and extraction, were deemed unsuitable for this case. Instead, a tailored approach combining forward and reverse electrosurgical resection was employed to excise the lesion (14) systematically. Given the attenuated myometrial thickness at the scar site, sole reliance on electrocautery would have significantly increased the risk of uterine perforation. To mitigate this, the fibroid base was carefully dissected both bluntly and sharply from the scar tissue when approaching this critical area, followed by meticulous removal of residual tissue using alternating resection techniques. This strategic approach ensured the safe and successful completion of the procedure.

The surgical procedure consumed 22,000 mL of distension fluid with an operative duration of 1 h and 45 min, both of which exceeded conventional thresholds for hysteroscopic surgeries (15, 16). Such parameters theoretically elevate the risk of fluid overload complications, particularly dilutional hyponatremia (17). However, the intramural fibroid’s unique localization within the cervical canal created a distinctive fluid dynamic: most irrigation fluid was lost through cervical canal leakage rather than being retained in the uterine cavity. This anatomical particularity significantly limited systemic fluid absorption, thereby mitigating the risk of electrolyte imbalance.

Intraoperative safety was ensured through rigorous monitoring protocols, real-time quantification of fluid deficit (input/output balance showing 22,000 mL infused versus 21,000 mL recovered), serial arterial blood gas analyses (performed twice during the procedure) demonstrating maintained sodium concentration within physiological ranges, and prophylactic administration of diuretics to counteract potential fluid overload. The paradoxical advantage of the fibroid’s cervical location warrants emphasis. While technically challenging due to obscured visualization and prolonged operative time, the predominant extrauterine fluid egress created an inherent safety mechanism. This explains the successful outcome despite the unusually high fluid volume and extended surgical duration. The case demonstrates that when accompanied by meticulous monitoring, hysteroscopic resection of cervically located intramural fibroids can be performed safely even when exceeding standard fluid parameters.

## 4 Conclusion

For large fibroids protruding from the scar site of cesarean section into the uterine cavity, hysteroscopy is the least damaging surgical method if operated by experienced doctors. However, strict calculation of fluid absorption and monitoring of blood gas analysis are required during the operation. Even if the surgical time and volume of dilation fluid exceed the conventional dosage, hysteroscopic surgery can still safely remove the fibroid. The drawback is that the lack of pelvic computed tomography (CT) or magnetic resonance imaging (MRI) examination before surgery resulted in insufficient preoperative assessment of surgical difficulty.

## Data Availability

The original contributions presented in the study are included in the article/Supplementary material, further inquiries can be directed to the corresponding authors.
