# Supplementary figures and images for: Hysteroscopic surgery for treating intramural fibroids at the cesarean scar area: a case report and literature review
Source: Front Med (Lausanne). 2025 Sep 9;12:1632322. doi: 10.3389/fmed.2025.1632322 (PMC12454310; doi:10.3389/fmed.2025.1632322)

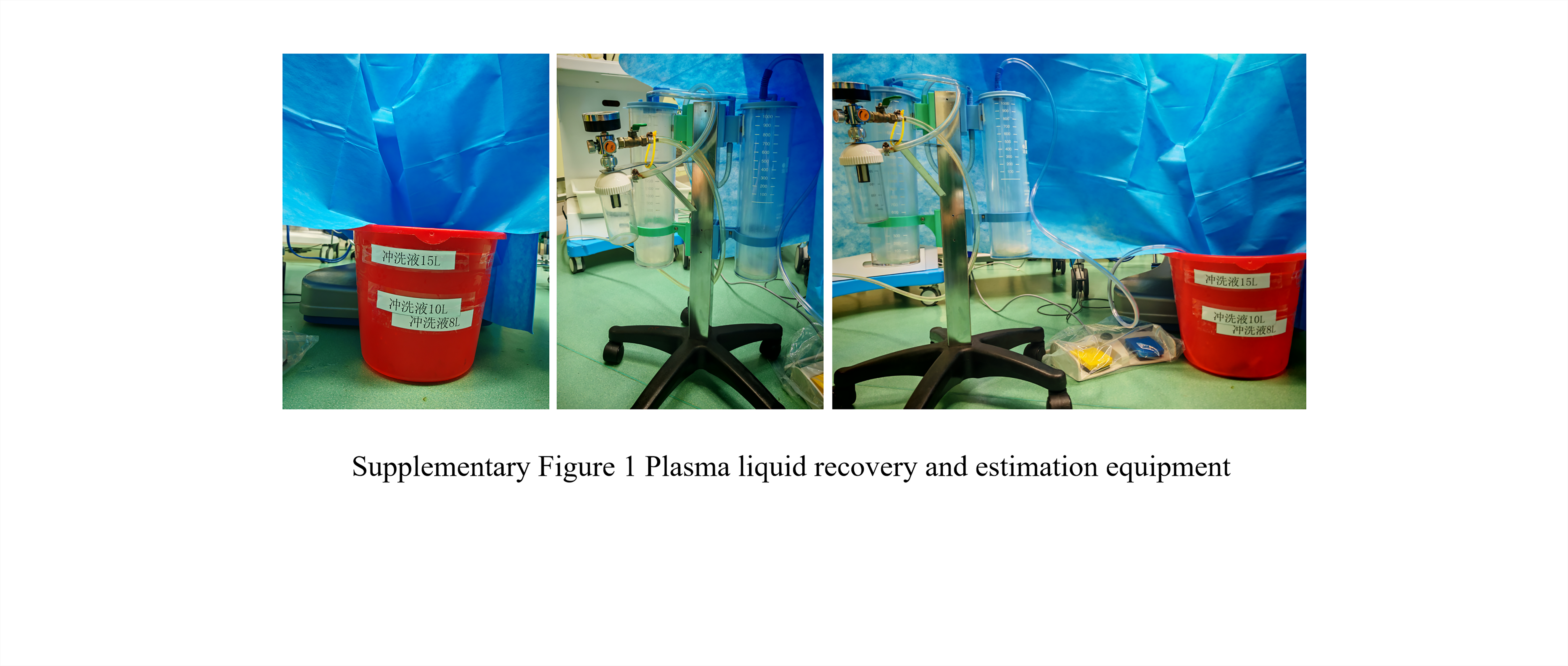

Supplement: Supplementary file 1 [file Image_1.tif]
